# SMARCA4‐deficient undifferentiated tumor that responded to chemotherapy in combination with immune checkpoint inhibitors: A case report

**DOI:** 10.1111/1759-7714.14547

**Published:** 2022-07-02

**Authors:** Takahiro Utsumi, Yohei Taniguchi, Yuri Noda, Mari Fukai, Kayoko Kibata, Tomohiro Murakawa

**Affiliations:** ^1^ Department of Thoracic Surgery Kansai Medical University Osaka Japan; ^2^ Department of Pathology and Laboratory Medicine Kansai Medical University Osaka Japan; ^3^ Department of Thoracic Oncology Kansai Medical University Osaka Japan

**Keywords:** lung neoplasms, mediastinal diseases, non‐small‐cell lung carcinoma, pleural effusion, respiratory distress syndrome

## Abstract

Thoracic SMARCA4‐deficient undifferentiated tumors are a new type of neoplasm that commonly occur in the mediastinum, progress rapidly, and show a poorer prognosis. We report a case of thoracic SMARCA4‐deficient undifferentiated tumor in the right thoracic cavity in a patient with a history of heavy smoking and presenting with respiratory distress and hemoptysis. Imaging showed pleural effusion and thickening. A diagnostic right pleural biopsy yielded multiple white nodules and pale bloody pleural effusion accumulated in the right thoracic cavity. Histopathologically, the tumor cells were large, some exhibited rhabdoid cytology, and they were surrounded by an infiltration of inflammatory cells. These tumor cells were negative for SMARCA4, p40, NUT, and claudin‐4, leading to establishing a diagnosis of thoracic SMARCA4‐deficient undifferentiated malignancy. We treated the patient with atezolizumab, carboplatin, and nab‐paclitaxel. The patient achieved stable disease at 7 months during this study. Although there is no standard treatment of this disease, our reported treatment may contribute to improved prognosis, requiring further research.

## Graphical abstract

Thoracic SMARCA4‐deficient undifferentiated tumors are a new type of neoplasm that commonly occur in the mediastinum and are associated with poor prognosis. To date, there is no standard treatment of thoracic SMARCA4‐deficient undifferentiated tumors. We report a case of thoracic SMARCA4‐deficient undifferentiated tumor with hemoptysis. We treated the patient with atezolizumab, carboplatin, and nab‐paclitaxel, which resulted in achieving stable disease for 7 months. Our reported treatment may contribute to improved prognosis, which requires further evaluation.

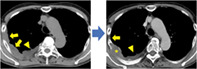

## INTRODUCTION

Thoracic SMARCA4‐deficient undifferentiated tumors have recently been recognized. SMARCA4 deficiency is also found in non‐small‐cell lung cancer. However, it is classified as an independent disease due to differences in tissue morphology, ribonucleic acid expression, and prognosis. SMARCA4 deficiency was first defined in the 5th edition of the World Health Organization classification of tumors.^1^ It often occurs in the mediastinum of male heavy smokers. To date, there is no established treatment of this disease, and its prognosis is poor at 4–7 months.^2^

Herein, we report the case of a patient with a right thoracic SMARCA4‐deficient undifferentiated tumor who underwent chemotherapy and achieved stable disease for 7 months.

## CASE REPORT

**FIGURE 1:**
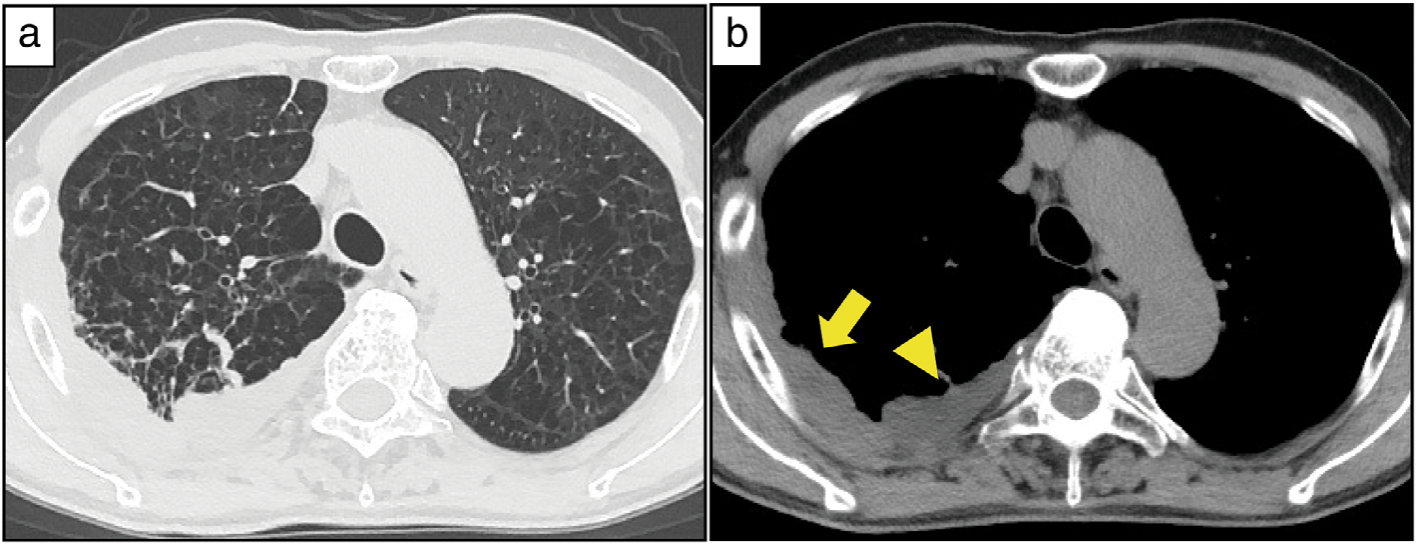
Preoperative computed tomography images. (a) Significant emphysema observed in the pulmonary window setting. (b) Right pleural thickening (yellow arrow) and effusion (yellow arrowhead) observed in the mediastinal window setting

A 72‐year‐old man with an 80‐pack year smoking history presented with respiratory distress and bloody sputum. Computed tomography revealed significant emphysema, right pleural thickening, and pleural effusion (Figure 1a,b). Tumor marker tests were negative. A diagnostic right pleural biopsy yielded several white nodules and1600 ml of pale bloody pleural effusion accumulated in the right thoracic cavity.

**FIGURE 2:**
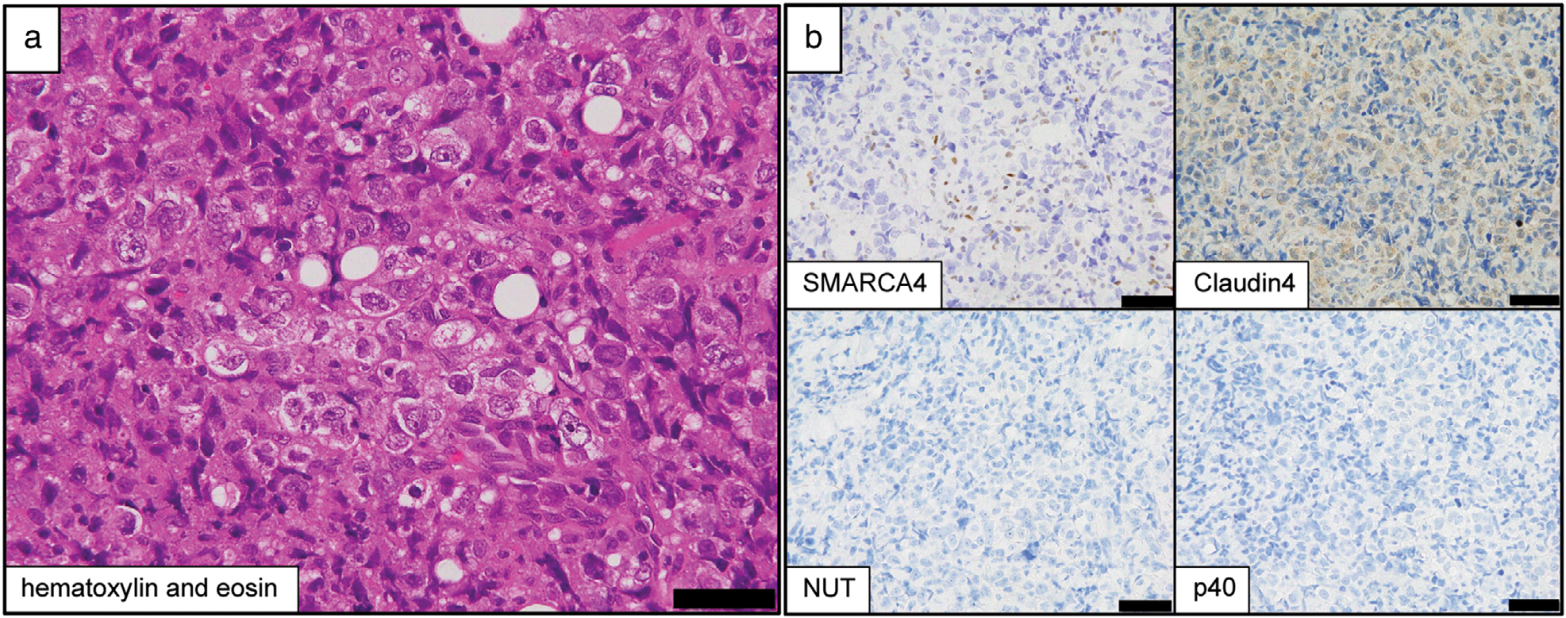
Pathological findings. (a) Large tumor cells with acidophilic cytoplasm are observed in sheets, and multinucleated cells and mitotic figures are also seen. Numerous inflammatory cells are seen infiltrating the surrounding area (hematoxylin and eosin staining). Scale bar = 50 μm. (b) Immunohistochemical staining results show that the tumor cells are negative for SMARCA4, claudin‐4, NUT, and p40. Scale bar = 50 μm

Histopathologically, large tumor cells with inflammatory cell infiltrates were found, some of which showed rhabdoid cytology. We initially suspected malignant pleural mesothelioma. However, since the cells were poorly differentiated and showed rhabdoid cytology, we performed immunohistochemical staining for makers for epithelial tumors such as lung cancer. Immunohistochemical staining showed that the tumor cells were negative for p40, NUT, claudin‐4, and SMARCA4 (Figure 2a,b). We subsequently diagnosed the patient with a thoracic SMARCA4‐deficient undifferentiated tumor.

**FIGURE 3:**
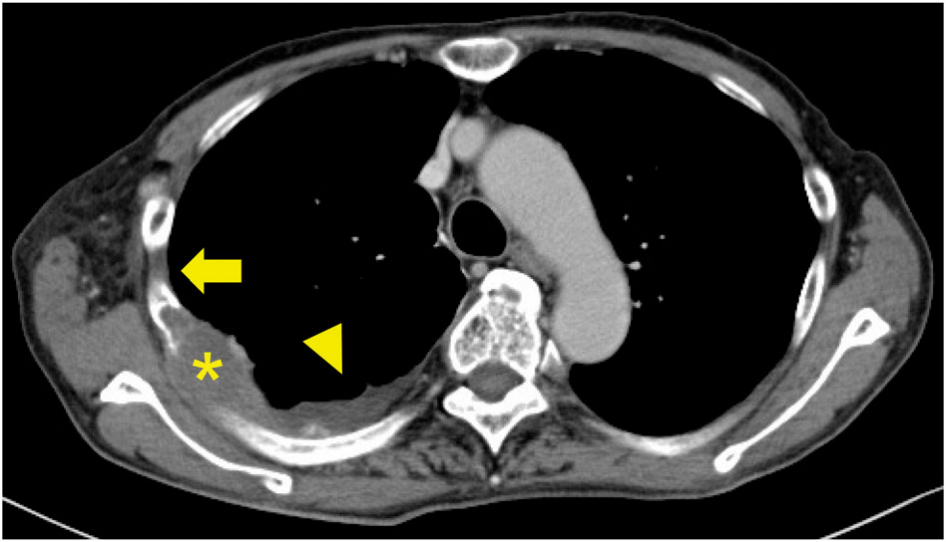
Computed tomography images after treatment. Thoracic SMARCA4‐deficient undifferentiated tumor showing osteolytic changes in the ribs (asterisk) is noted. However, pleural thickening (yellow arrow) disappears and pleural effusion (yellow arrowhead) decreases in the mediastinal window setting.

Chemotherapy with atezolizumab, carboplatin, and nab‐paclitaxel did not improve the symptoms, including respiratory distress and bloody sputum. Imaging showed bone infiltration, but pleural thickening disappeared and pleural effusion decreased (Figure 3). We therefore determined the patient to have stable disease and he survived for 7 months.

## DISCUSSION

*SMARCA4* is a gene involved in the switch/ sucrose‐nonfermenting (SWI/SNF) chromatin‐remodeling complex. Transcription is regulated by the polycomb repressor complex (PRC). Moreover, the SWI/SNF complex acts as a tumor suppressor. When *SMARCA4* is deficient, the PRC of the SWI/SNF complex is not suppressed, leading to tumor progression.^2^

Immunostaining can help distinguish thoracic SMARCA4‐deficient undifferentiated tumors from SMARCA4‐deficient lung cancer or proximal‐type epithelioid sarcoma.^3^ In general, thoracic SMARCA4‐deficient undifferentiated tumors are positive for CD34, SOX2, and SALL4, and negative for claudin‐4^4^ and have a simultaneous deficiency of SMARCA4 and SMARCA2, whereas SMARCA4‐deficient lung cancer has a less simultaneous deficiency. Negative p40 and NUT results are useful for establishing auxiliary diagnoses.^2^

There is no standard treatment of thoracic SMARCA4‐deficient undifferentiated tumors. However, enhancers of zeste homolog 2 inhibitors that suppress PRC and tumor progression, which act as a catalytic subunit of PRC, are in clinical trials.^4^

Regarding advanced non‐small‐cell lung cancer, the combination of anti‐PD‐1/PD‐L1 antibody and a cytotoxic drug has been shown to be effective regardless of PD‐L1 expression in tumor cells. Furthermore, the synergistic effect of anti‐PD‐1/PD‐L1 antibody and anti‐angiogenic agents has also been demonstrated.^5^, ^6^ It has been reported that the therapeutic effect was obtained using the anti‐PD‐L1 antibody in thoracic SMARCA4‐deficient undifferentiated tumors, regardless of the degree of PD‐L1 expression.^7^, ^8^ Although the efficacy of anti‐angiogenic agents in sarcoma has not been established, it has been reported that treatment with atezolizumab, bevacizumab, carboplatin, and paclitaxel was effective in thoracic SMARCA4‐deficient undifferentiated tumors.^9^ Our patient was treated with atezolizumab, carboplatin, and nab‐paclitaxel, excluding anti‐angiogenic agents for hemoptysis, and achieved stable disease for 7 months.

More patients with thoracic SMARCA4‐deficient undifferentiated tumors might be seen in clinical practice, therefore we should consider thoracic SMARCA4‐deficient undifferentiated tumors when identifying pathological features such as large tumor cells showing rhabdoid cytology with infiltration of inflammatory cells assessed by hematoxylin and eosin staining. There have been few reports on the effectiveness of immune checkpoint inhibitor combination therapy in thoracic SMARCA4‐deficient undifferentiated tumors, which may be a new treatment option.

## CONCLUSIONS

A patient with thoracic SMARCA4‐deficient undifferentiated tumor who was treated with chemotherapy and immune checkpoint inhibitors displayed 7 months of stable disease. Further research is necessary to establish a standardized treatment strategy. There have been a few reports of treatment with immune checkpoint inhibitors, and more cases need to be accumulated to evaluate whether this treatment is effective.

## AUTHOR CONTRIBUTIONS

T.U.: conceptualization, data curation, investigation, and writing of the original daft. Y.T. and T.M.: supervision. N.Y.: pathological diagnosis. M.F. and K.K.: treatment.

## FUNDING INFORMATION

This research did not receive any specific grant from funding agencies in the public, commercial, or not‐for‐profit sectors.

## CONFLICT OF INTEREST

None declared.
